# Effect of Polyhydroxyalkanoate (PHA) Concentration on Polymeric Scaffolds Based on Blends of Poly-L-Lactic Acid (PLLA) and PHA Prepared via Thermally Induced Phase Separation (TIPS)

**DOI:** 10.3390/polym14122494

**Published:** 2022-06-19

**Authors:** Francesco Lopresti, Antonio Liga, Elisa Capuana, Davide Gulfi, Claudio Zanca, Rosalinda Inguanta, Valerio Brucato, Vincenzo La Carrubba, Francesco Carfì Pavia

**Affiliations:** 1 Department of Engineering, University of Palermo, RU INSTM, Viale delle Scienze, 90128 Palermo, Italy; antonio.liga@community.unipa.it (A.L.); elisa.capuana@unipa.it (E.C.); davide.gulfi@community.unipa.it (D.G.); claudio.zanca@unipa.it (C.Z.); rosalinda.inguanta@unipa.it (R.I.); valerio.brucato@unipa.it (V.B.); francesco.carfipavia@unipa.it (F.C.P.); 2 ATeN Center, University of Palermo, Viale delle Scienze, 90128 Palermo, Italy; 3 Consorzio Universitario di Caltanissetta, Corso Vittorio Emanuele 92, 93100 Caltanissetta, Italy

**Keywords:** tissue engineering, biopolymer blends, porous structures, scaffold, thermally induced phase separation

## Abstract

Hybrid porous scaffolds composed of both natural and synthetic biopolymers have demonstrated significant improvements in the tissue engineering field. This study investigates for the first time the fabrication route and characterization of poly-L-lactic acid scaffolds blended with polyhydroxyalkanoate up to 30 wt%. The hybrid scaffolds were prepared by a thermally induced phase separation method starting from ternary solutions. The microstructure of the hybrid porous structures was analyzed by scanning electron microscopy and related to the blend composition. The porosity and the wettability of the scaffolds were evaluated through gravimetric and water contact angle measurements, respectively. The scaffolds were also characterized in terms of the surface chemical properties via Fourier transform infrared spectroscopy in attenuated total reflectance. The mechanical properties were analyzed through tensile tests, while the crystallinity of the PLLA/PHA scaffolds was investigated by differential scanning calorimetry and X-ray diffraction.

## 1. Introduction

Over the recent years, large technological and scientific interest have dealt with the possibility of controlling polymer foams products to be employed as scaffolds for tissue engineering applications [1,2,3]. Many techniques have been developed to produce porous tissue engineering scaffolds, such as porogen leaching [4,5], freeze drying [6,7], 3D printing [8,9,10], electrospinning [11,12,13], thermally induced phase separation (TIPS) [14,15,16] and any possible combinations of these [17]. Among the listed techniques, TIPS is one of the most efficient due to its ease of implementation and potential capability to produce highly porous scaffolds with tunable properties [15,17]. Different parameters can be considered to obtain the required properties, such as the polymeric system (including blends), polymer concentration, solvent and nonsolvent system, and cooling rate [18]. Adjusting such parameters allows for fine control of significant scaffold properties, including morphology, pore size, degree of interconnected pores, biodegradability and mechanical properties [15]. In this context, there is a growing interest in hybrid biopolymeric porous scaffolds composed of both synthetic and natural biopolymers exhibiting high mechanical properties and the ability to support cell attachment and proliferation [19,20,21,22,23].

Poly-L-lactic acid (PLLA) can be considered one of the most interesting synthetic biopolymers for tissue regeneration due to its interesting mechanical properties and good processability [24,25,26]. However, like other synthetic polymers, PLLA is hydrophobic, thus leading to poor cell affinity by hindering cell adhesion [27,28]. To overcome these limits, PLLA may be filled with organic/inorganic nanoparticles and/or coupled with other synthetic or natural polymers [29,30,31,32,33]. Generally, natural biopolymers show enzyme-controlled degradability, good biocompatibility, low inflammatory potential, high chemical versatility and similarities with the extracellular matrix [34,35]. For this reason, a variety of natural biopolymer-based scaffolds composed of alginate, dextran, hyaluronic acid, kefiran and chitosan, among others, have been recently used in tissue engineering and regenerative medicine strategies due to their excellent biocompatibility combined with their potential biodegradability [11,34,36,37,38,39]. Polyhydroxyalkanoates (PHAs) are a family of biodegradable polyesters intracellularly produced by many microorganisms as carbon and energy storage compounds under unbalanced growth conditions [40,41]. The biocompatibility and applications of PHA in tissue engineering have been studied by many research groups due to their high biocompatibility as well as cell growth and proliferation capacity [42,43,44,45,46,47,48].

In this work, for the first time, PLLA scaffolds in blend with PHA up to 30 wt% were prepared by the TIPS method starting from ternary solutions. The scaffolds were characterized in terms of the surface chemical properties carried out by Fourier transform infrared spectroscopy in attenuated total reflectance (FTIR-ATR). The microstructure of the hybrid mats was analyzed by scanning electron microscopy (SEM) and related to the wettability of the scaffolds evaluated through water contact angle (WCA) measurements. The mechanical properties were analyzed through tensile tests, while the crystallinity of the PLLA/PHA scaffolds was investigated by differential scanning calorimetry (DSC) and X-ray diffraction (XRD).

## 2. Materials and Methods

### 2.1. Materials

Highly crystalline poly-L-lactic-acid (PLLA, Resomer^®^ L 209 S, Inerent Viscosity 2.6–3.2 dL/g by Evonik specifications, Evonik, Essen, Germany) was supplied by Boehringer Ingelheim Pharma KG Ingelheim am Rhein, Germany. A commercial grade of PHA was provided by Sigma Aldrich, Munich, Germany, as well as 1,4-dioxane (ACS grade, purity > 99%), used as solvent.

### 2.2. Foams Preparation

A homogeneous ternary solution of PLLA or PLLA/PHA blends (PLLA/PHA 90/10, 80/20 and 70/30 wt/wt), dioxane, and water was prepared, with constant dioxane to water weight ratio of 87/13, based on previous literature studies on similar systems [49]. The total concentration of the polymer phase in the solvent mixture was 4 wt%. The solution, initially kept at 60 °C, was hot poured into an aluminum disc-shaped sample holder with a diameter of 60 mm and a thickness of 2.5 mm. The temperature was then suddenly lowered to a well-defined value (30 °C or 35 °C) for 10 min by pool immersion of the sample holder into a thermostatic water bath. Then, a quench by pool immersion in an ethyl alcohol bath at a temperature of −20 °C for 10 min was performed to freeze the as obtained structure. For direct quench samples (DQ), the sample holder was directly immersed in a −20 °C bath for 15 min. Then, the frozen samples were immersed in distilled water at room temperature and rinsed abundantly for 12 h in order to remove any trace of dioxane. Finally, the samples were dried under vacuum in an oven at 37 °C for 24 h.

### 2.3. Morphological Analysis

The morphology of the scaffolds was evaluated by scanning electron microscopy (SEM-FEI QUANTA 200F, FEI, Hillsboro, OR, USA). Nitrogen-fractured scaffolds were attached by using adhesive carbon tape on an aluminum stub. Before the analysis, the samples were sputter-coated with gold for 60 s under an argon atmosphere by using a Sputtering Scancoat Six (Edwards Laboratories, Milpitas, CA, USA) in order to avoid electrostatic charge during the test [11].

### 2.4. Foams Porosity

The porosity of PLLA/PHA scaffolds was calculated as the reciprocal of the ratio between the apparent density of the scaffold and the non-porous polymeric material density by using Equation (1).

(1) $$Porosity\;\left(\%\right)=\left(1-\frac{\rho_{scaffold}}{\rho_{bulk}}\right)\times 100\;\;\;\;\;\;\;\;\;$$

where $\rho_{scaffold}$ is the apparent density of the foam while the bulk density ($\rho_{bulk}$) of PLLA/PHA foams was evaluated by using a helium pycnometer (Pycnomatic ATC from Thermo Fisher Scientific, Waltham, MA, USA). For each sample, at least ten measurements were carried out, and the average value was then recorded. In all the cases, the standard deviations were lower than 0.01 g/cm^3^.

### 2.5. FT-IR/ATR Analysis

FT-IR/ATR analysis (FT-IR/NIR Spectrum 400 spectrophotometer from Perkin-Elmer Inc., Wellesley, MA, USA) was performed to investigate the sample chemical surface properties. For each sample, 4 accumulations scans with a resolution of 4 cm^−1^ were collected in the range of 4000–400 cm^−1^.

### 2.6. Differential Scanning Calorimetry

A differential scanning calorimeter (Setaram Instrumentation, Caluire, France, model DSC131) was used to investigate the calorimetric properties of the scaffolds. The analysis was carried out by heating the samples, whose weight was about 5 mg, from room temperature to 200 °C at 10 °C/min heating rate under nitrogen flow.

PLLA and PHA crystallinity degree (χ) were calculated according to Equation (2) [11]:

(2) $$\chi_{i}\;\left(\%\right)=\frac{∆H_{m}-∆H_{\mathrm{cc}}}{∆H^{0}{}_{i\;}\times {\;X}_{i}}\times 100$$

where $∆H_{\mathrm{cc}}$ and $∆H_{m}$ are the cold crystallization and melting enthalpy of the samples, respectively. $X_{i}$ is the weight fraction of PLA or PHA, and $∆H^{0}{}_{i\;}$ is the melting enthalpy of 100% crystalline PLLA or PHA equal to 93.7 J/g [11] and 145 J/g [50], respectively.

### 2.7. X-ray Diffraction

X-ray diffraction patterns were collected by using a RIGAKU diffractometer (model: D-MAX 25600 HK, Rigaku, Tokyo, Japan). All diffraction patterns were obtained in the 2θ range from 5° to 60° by means of copper Kα radiation (λ = 1.54 Å) with the following setup conditions: tube voltage and current of 40 kV and 30 mA, respectively, scan speed of 4°/min with a sampling of 0.004°.

### 2.8. Water Contact Angle Measurements

The static contact angles test was performed using an FTA 1000 (First Ten Ångstroms, Cambridge, UK) instrument using distilled water (DW) as fluid. In particular, a droplet of DW (~4 μL) was dropped on the scaffold, and the images were taken after 10 s from the DW deposition. At least 7 spots of each sample were measured, and the average value was taken.

### 2.9. Mechanical Properties

A laboratory dynamometer (Instron model 3365, Instron, High Wycombe, UK) equipped with a 1 kN load cell was used to perform the compressive mechanical measurements on cylindrical specimens (2.5 mm height and 10 mm diameter).

The scaffolds were cut off from the entire disc by using a punch with a diameter of 10 mm. The compressive tests were carried out at 1 mm/min up to 2 mm of displacement. The initial height of the samples was measured before the test. Seven samples were tested for each material, and the average values of the mechanical parameters were reported with their standard deviations.

### 2.10. Statistical Analysis

Statistical analyses of the data were performed through one-way analysis of variance, and when applicable, data were compared using the Student’s *t*-test. *p*-value < 0.05 was considered statistically significant.

## 3. Results and Discussion

### 3.1. Morphology of PLLA/PHA Scaffolds as a Function of PHA Content and Thermal History

**Figure 1:**
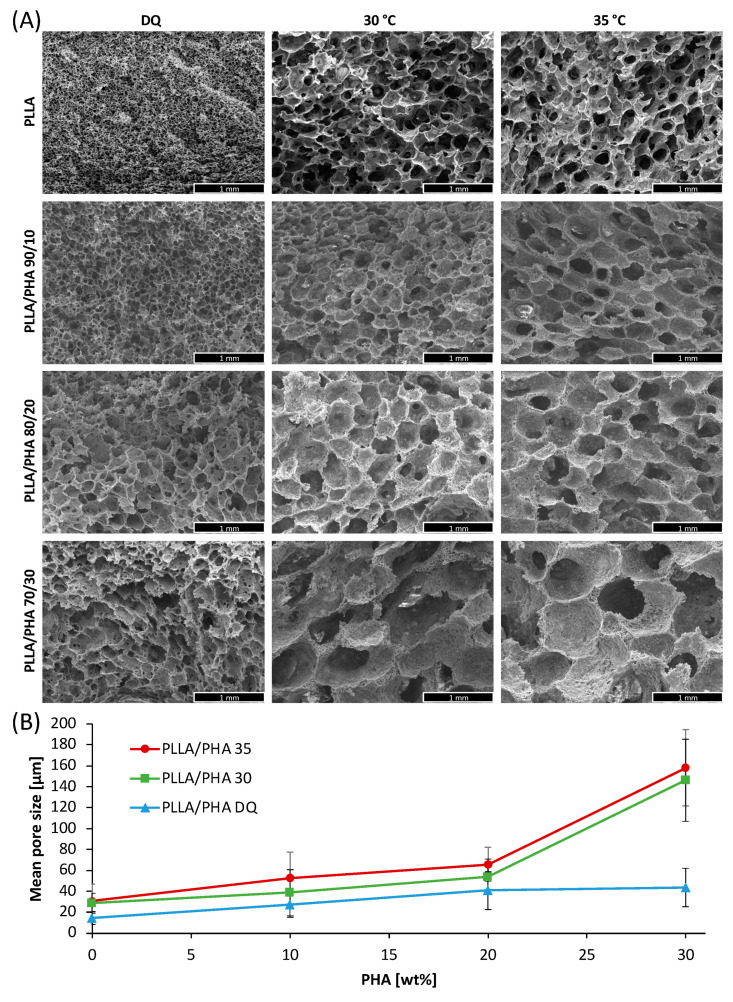
(**A**) SEM micrographs of PLLA/PHA foams prepared at different demixing temperatures; (**B**) mean pore size of the PLLA/PHA foams as a function of the PHA wt% in the blend. Scale bars are 1 mm.

Figure 1A represents the morphology of PLLA/PHA as a function of PHA content and the thermal history of the solutions. 

The first line of Figure 1A displays the morphology of pure PLLA scaffolds as a function of the processing temperature. More in detail, the porous structure obtained by direct quenching (DQ), without an intermediate cooling step, shows small, interconnected pores as already observed in previous work [51]. On the other hand, the PLLA samples obtained when keeping the solution at a temperature within the metastable range (i.e., 30 and 35 °C [51]) show bigger pores but lower connectivity.

As a matter of fact, for PLLA DQ samples, a spinodal decomposition seems to take place (witnessed by the interpenetration of the two phases). In this case, the solidification/precipitation of the polymer matrix occurs before water and dioxane are allowed to separate in two different phases, thus generating small nuclei. Conversely, for 30 and 35 °C, samples exhibit a typical morphology derived from a nucleation and growth mechanism, characterized by a bigger average pore size scaffolds and a lower degree of interconnection with respect to the PLLA DQ samples. This result is totally in agreement with a previous work [52] in which the cloud point curve for a similar system was obtained. Specifically, for a polymer concentration of 4 wt% with a dioxane/water ratio of 87/13 wt/wt, a cloud point at about 41 °C was detected [52].

When adding PHA to PLLA, the trend of the pore size of the scaffolds as a function of the processing temperature is similar to that observed for pure PLLA scaffolds. 

However, the pores morphology remarkably changes since the addition of even small amounts of PHA reflected into bigger pores than in the case of PLLA alone, regardless of the processing temperature. This is visible in Figure 1A lines 2-3-4 and in Figure 1B, reporting the mean pore size of the PLLA/PHA foams as a function of the PHA concentration in the blends. Interestingly, the increment of pore size as a function of the PHA concentration is more evident in the PLLA/PHA foams prepared at 30 and 35 °C.

The observed morphologies led one to suppose that the presence of PHA determines a lowering of the cloud point curve, leading to a bigger pore size with respect to pure PLLA scaffolds. From these data, and in agreement with a previous study concerning PLLA/PLA blended foams [53], it can be assumed that, at a fixed polymer concentration and dioxane/water ratio, the higher the PHA concentration, the lower the cloud point. This affirmation could be easily explained by inferring poor miscibility between the two polymers [54,55] and, consequently, an absence of interaction between them. It is well known that the cloud point is related to the total polymer concentration in the solution. Specifically, the lower the polymer concentration, the lower the cloud point [52]. In previous work, we showed that for an 87/13 dioxane/water ratio, the cloud point decreases from 40 to 32 °C when decreasing the polymer concentration from 4 to 2%. In our case, the concentration of the PLLA phase alone decreased from 4 to 2.8 % when adding PHA, thus leading to a reduction of the solution cloud point, coupled with the aforementioned increase in the average pore size. Unfortunately, it was not possible to evaluate the cloud points experimentally because of the intrinsic opacity of the solutions in the presence of PHA. It is, however, licit to hypothesize that, in the range of temperatures taken into consideration, PHA is abundantly over its cloud point. Otherwise, the morphology of the scaffolds would have been affected by PHA demixing, as shown in previous work [53].

However, the poor pores interconnection observed in PLLA/PHA 30 and 35 °C samples make these scaffolds unsuitable for tissue engineering purposes. Therefore, the other characterizations were carried out on PLLA/PHA DQ foams.

### 3.2. FTIR-ATR Analysis

To evaluate the effective inclusion of the PHA phase in PLLA/PHA scaffolds prepared via the TIPS approach, FTIR-ATR measurements were carried out on all the PLLA/PHA foams prepared via direct quenching.

**Figure 2:**
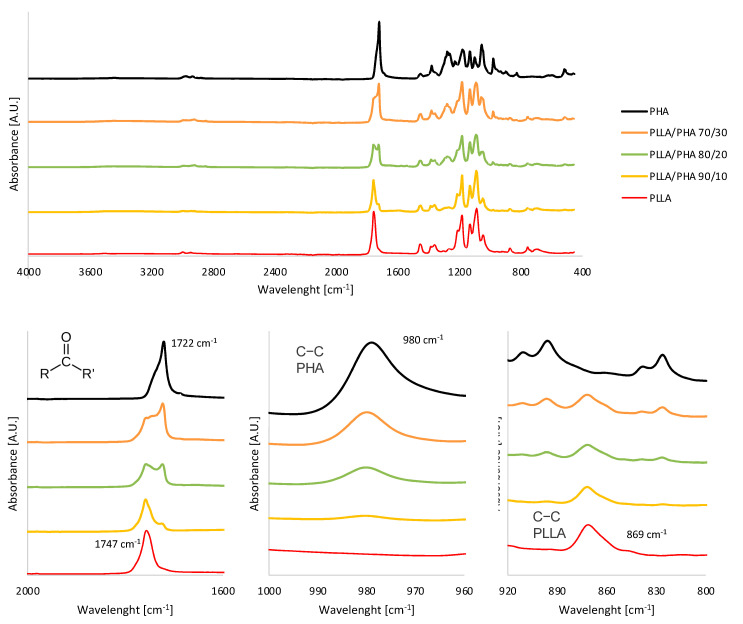
FTIR-ATR spectra of PLLA/PHA scaffolds as a function of the PHA concentration in the blend.

The ATR-FTIR spectrum of PLLA (Figure 2) showed several peaks usually attributed to this polymer, such as the carbonyl stretch at 1747 cm^−1^ (C=O, highlighted in Figure 2), the C–O stretch at 1180 cm^−1^, 1129 cm^−1^ and 1083 cm^−1^ and the OH bend at 1044 cm^−1^ [11]. The bands visible at 869 cm^−1^ highlighted in Figure 2 can be assigned to the C-C stretch of the PLLA crystalline phase [11]. 

The FTIR-ATR spectrum of PHA showed prominent peaks at 1726 cm^−1^ and 1223–1132 cm^−1^, denoting carbonyl (C=O, highlighted in Figure 2) and asymmetric C-O-C stretching vibration, respectively, characteristic for ester bonding found in PHA molecule [56,57]. Other adsorption bands visible at 980 cm^−1^ (highlighted in Figure 2) were assigned to C-CH_3_ stretching vibration, while the band at 3442 cm^−1^ denoted the C-H (asymmetric vibration) of PHA [57].

The PLLA/PHA hybrid scaffold spectra are clearly an overlapping of the single spectra of PLLA and PHA. More in detail, next to the carbonyl stretch of PLLA at 1747 cm^−1^, a band at 1722 cm^−1^ ascribable to the same functional group of PHA is clearly visible also in the PLLA/PHA scaffolds and rises upon increasing the PHA ratio in the blends. Similarly, the C-C stretch of the PHA phase at 980 cm^−1^ gradually disappears upon reducing the PHA ratio, while the peak ascribed to the C-C stretch of PLLA intensifies.

As expected, the characteristic peaks ascribable to the two polymeric phases are well distinguishable in the FTIR-ATR spectra of the blends, and the peaks absorbance related to the PHA phase increases upon incrementing the PHA ratio in the blends, thus ensuring the effective inclusion of PHA phase in the blend via TIPS. Moreover, the absence of band shifts indicates that this procedure did not activate the chemical reaction between PLLA and PHA.

### 3.3. Thermal and XRD Analysis 

**Figure 3:**
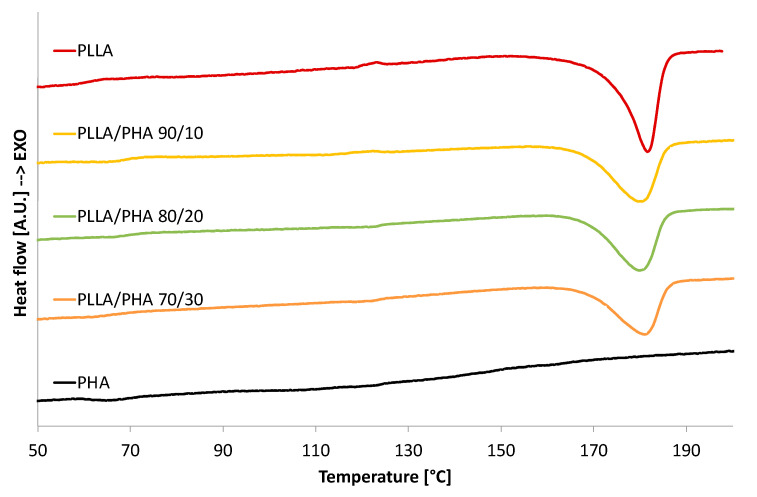
DSC thermograms of the PLLA/PHA foams.

Figure 3 displays the DSC thermograms obtained for all samples produced via direct quenching, while Table 1 reports the data derived from DSC analysis.

As expected, pure PLLA scaffold exhibits a glass transition around 66.5 °C, a cold crystallization peak around 123 °C, and an endothermic peak ascribable to the polymer melting centered around 182 °C. To consider the effect of the processing on the thermal properties of pure PHA, a pure PHA foam was prepared by using the same conditions used for pure PLLA foams. The thermogram of foamed PHA showed a small endothermic peak around 65.5 °C, ascribable to a glass transition. At higher temperatures, a broad endothermic peak in the temperature range of 90–160 °C was attributed to the melting of the crystalline phase of PHA.

The thermograms of PLLA/PHA foams revealed that the presence of PHA in the blend does not significantly influence the melting temperature of PLLA (which remains almost constant for all the investigated samples, Table 1). This suggests that the morphology of the PLLA crystals has not been altered by PHA in the blend.

From Figure 3, a reduction in the melting peak of PLLA can be noticed when the amount of PHA increases in the PLLA/PHA blends. On the other hand, the broad melting peak of PHA is not noticeable in the PLLA/PHA foams, thus suggesting that PLLA significantly reduced the crystallinity of the PHA component in the blends, as already reported in [54,58].

The crystallinity of the PLLA phase in the PLLA/PHA blends, reported in Table 1, is affected by the presence of PHA in the blends. More in detail, the crystallinity of the pure PLLA scaffold was nearly 70%. The addition of PHA led to a steplike decrease in crystallinity down to around 59% regardless of its concentration in the PLLA/PHA blend. This result was already observed in literature, and it was attributed to the ability of the PHA molecular chains to hinder the chain mobility of PLLA [59] and vice versa since the melting peak of PHA is not visible in the PLLA/PHA blends.

Regrettably, it was not possible to evaluate the miscibility of the PLLA/PHA blends from DSC analysis since their T_g_s were very close [54]; however, according to scientific literature, it can be presumed low miscibility among the two polymer phases due to the relatively high molecular weight of PLLA used in this work [54,55].

**Figure 4:**
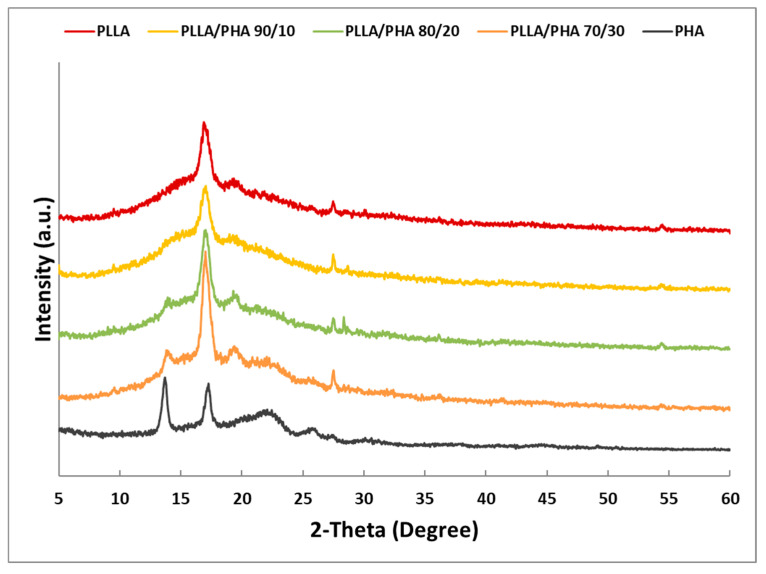
XRD patterns of the PLLA/PHA foams.

In Figure 4, the X-ray diffraction patterns carried out on PLLA/PHA scaffolds at increasing concentrations of PHA were reported. In addition, XRD patterns of the pure PLLA and PHA polymers were added to better compare with scaffold blends. In the PLLA pattern, the two characteristic main peaks at 2 theta 16.8° and 19.5° were found [60]. For pure PHA, according to literature data [61], it can be observed the presence of two main narrow peaks at 2-theta 13.5° and 17.2° and two broad peaks for 2-theta 21.6° and 25.7°.

The main peak of PHA at 13.5° appears with increasing polymer concentration in the blend, and it is more noticeable for 80/20 and 70/30 PLLA/PHA blends. The same behavior was found for the broad band at 21.6° of the PHA, especially for the 70/30 blend. Nevertheless, no significant modification to PLLA in blends was observed, considering that its main diffraction peaks at 16.8° were not influenced by the presence of PHA. Doubtless, all blend samples showed a low crystallinity of their PHA component characterized by low intensity and high width of diffraction peaks. These results suggest that PHA is able to crystallize also in blend with PLLA even if it was not detected via DSC analysis, probably because of the low melting enthalpy of the pure PHA.

### 3.4. Porosity and Wettability

**Figure 5:**
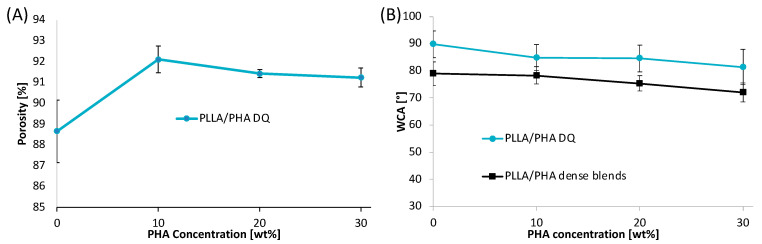
(**A**) Porosity and (**B**) water contact measurements of PLLA/PHA scaffolds as a function of the PHA content in the PLLA/PHA blend.

The scaffold porosity is a key parameter for tissue engineering applications. In this work, the scaffold porosity was calculated as the reciprocal of the ratio between the apparent density of the scaffold and the non-porous polymeric material density by using expression (2). The porosity values of the scaffolds, in the range of 86.5–92%, were found to be almost not affected by the PLLA/PHA blend composition (Figure 5A).

The surface wettability was analyzed to evaluate the hydrophilic/hydrophobic character of the scaffolds through water contact angle (WCA) measurements (Figure 5B). The wettability performance of porous scaffolds is strongly dependent on the chemical properties of the polymer matrix but also on the surface topographical properties of the foam. To remove the effect of the surface roughness and evaluate the effect of the blend chemical properties only, the WCA tests were also performed on sintered scaffolds that will be called “dense blends”. Results revealed that dense PLLA showed a water contact angle of 78.9° that remains almost constant at the lowest PHA concentration, i.e., 10 wt%, and then gradually decreases down to 71.9° for PLLA/PHA 70/30 dense blend. This result can be likely ascribed to the higher wettability of PHA if compared to PLLA [62].

The WCA values of PLLA/PHA foams showed a trend parallel to that of the dense blends but shifted at higher values of about 10°. To clarify these results, it may be considered that the wettability of polymeric blends is strongly dependent on the chemical properties of the materials [63] and also on the surface topographical properties. In fact, according to Wenzel’s theory of surface wetting [64], as the surface roughness increases, the water contact angle increases proportionally to the ratio of the real rough surface area to the projected perfectly smooth surface. Therefore, it is not surprising that the porous structure exhibits higher WCA values higher than that of dense blends and that the shift amongst the curves remains of the same order of magnitude regardless of the PLLA/PHA weight ratio.

### 3.5. Mechanical Properties of PLLA/PHA Scaffolds

**Figure 6:**
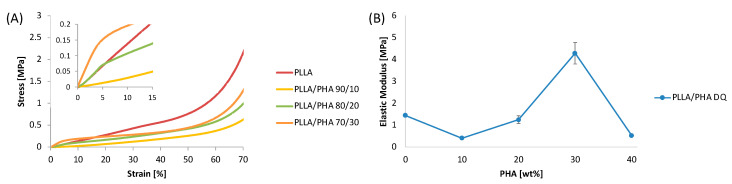
(**A**) Representative stress–strain curves of PLLA/PHA DQ foams; (**B**) Elastic modulus as a function of the PHA content in the PLLA/PHA DQ foams. Values of elastic modulus are given as means ± SD of *n* = 5 samples.

Figure 6A displays the representative stress–strain curves of PLLA/PHA scaffolds cooled via direct quench. The insert in Figure 6A highlight that all the samples presented an initial region (up to 5% of strain) characterized by a linear-elastic region probably related to the bending of the pore walls. Then, a transition region can be observed and associated with the establishment of a permanent plastic deformation [65]. The last region, after 60% of strain, depicts a steep growth of the stress owing to the densification of the foams induced by the pore walls collapse that fill the void of the porous structures.

The elastic moduli of the samples are summarized in Figure 6B as a function of the PHA concentration. PLLA scaffolds displayed an E value of nearly 1.5 MPa. The addition of 10 wt% of PHA to PLLA induced a steep decrease in the elastic modulus that can be mainly explained by the porosity increment observed for the PLLA/PHA 90/10 DQ samples and by the reduction of the PLLA crystallinity. In fact, the porosity values for PLLA DQ and PLLA/PHA 10% DQ were 88.6% and 92.1%, respectively, while the crystallinity decreased from around 70% down to 58%, while relatively low differences in the pore size distribution were observed via SEM. Upon increasing the PHA concentration in the PLLA/PHA DQ foams, a steep increment of the E value was recorded. In particular, at the highest PHA concentration, i.e., PLLA/PHA 70/30DQ samples, the elastic modulus was found to be equal to 4.3 MPa, almost three times higher than that of PLLA foams. These results can be likely rationalized by invoking a reinforcing action due to PHA since porosity, pore size distribution and PLLA crystallinity changes induced by the presence of PHA are very low if compared to the elastic modulus variations. The strengthening action of PHA can be ascribed to the presence of small fine dispersion of PHA crystals, detected via XRD analysis, performing as a reinforcing agent for the PLLA matrix [54].

## 4. Conclusions

In this work, the physical and surface chemical properties of hybrid scaffolds prepared via TIPS and composed of a blend of PLLA with PHA were evaluated. The morphological investigations revealed an increase in the scaffolds’ pore size upon increasing the processing temperature and the PHA concentration. The spectroscopic analysis confirmed the effective formation of the PLLA/PHA blend. The DSC thermograms of the scaffolds highlighted that PHA reduces the PLLA crystallinity while still maintaining values near 60%. XRD analysis confirmed the semicrystalline structure of pure PLLA and PHA scaffolds and that the crystallinity of PHA is dramatically reduced when blended with PLLA. The addition of PHA produced scaffolds exhibiting a comparable porosity and a slightly higher wettability if compared to neat PLLA scaffolds. The elastic moduli of the PLLA/PHA scaffolds containing the highest PHA concentration, i.e., 30 wt%, were found to triplicate if compared to the pure PLLA scaffold, thus demonstrating a reinforcing action of PHA on PLLA. The results obtained in this research may be instructive for designing processes of fabrication of PLLA-based hybrid porous scaffolds via TIPS. Moreover, this work may be considered a preliminary study for further experimental biological investigations such as cell adhesion, proliferation, infiltration and differentiation.

## Figures and Tables

**Table 1:** Melting temperature and melting enthalpy of PLLA-PHA samples.

Sample	T_g_ (°C)	T_cc_ (°C)	T_m_ (°C)	ΔH_m_ (J/g)	X_PLLA_ (%)	X_PHA_ (%)
PLLA/PHA 100/0	66.43	123.51	181.61	65.42	69.82	-
PLLA/PHA 90/10	66.73	122.95	180.43	48.97	58.07	-
PLLA/PHA 80/20	66.35	-	179.68	44.33	59.14	-
PLLA/PHA 70/30	64.87	-	181.12	39.19	59.76	-
PLLA/PHA 0/100	65.52	-	122.82	20.02	-	13.80

## Data Availability

The data presented in this study are available on request from the corresponding author.
